# Development of the Japanese version of the Minneapolis-Manchester Quality of Life Survey of Health - Adolescent Form (MMQL-AF) and investigation of its reliability and validity

**DOI:** 10.1186/s12955-014-0127-y

**Published:** 2014-08-15

**Authors:** Makiko Koike, Hiroki Hori, Takeshi Rikiishi, Akira Hayakawa, Naoko Tsuji, Tsukasa Yonemoto, Hideko Uryu, Eisuke Matsushima

**Affiliations:** Section of Liaison Psychiatry and Palliative Medicine, Graduate School of Tokyo Medical and Dental University, 1-5-45 Yushima, Bunkyo-ku, Tokyo 113-8519 Japan; Graduate School of Psychology, Mejiro University, 4-31-1 Nakaochiai, Shinjuku-ku, Tokyo 161-8539 Japan; Department of Medical Education/Pediatrics, Mie University Graduate School of Medicine, 2-174 Edobashi, Tsu-shi, Mie 514-8507 Japan; Department of Pediatrics, Tohoku University School of Medicine, 1-1 Seiryou-machi, Aoba-ku, Sendai–shi, Miyagi 980-8574 Japan; Department of Pediatrics, Kobe University Graduate School of Medicine, 7-5-1 Kusunokicho, Chuo-ku, Kobe-shi, Hyogo 650-0017 Japan; Niizashiki Central General Hospital, Department of Palliative Medicine, 1-7-2 Tohoku, Niiza-shi, Saitama 352-0001 Japan; Division of Orthopedic Surgery, Chiba Cancer Center, 666-2 Nitona-cho, Chuo-ku, Chiba 260-8717 Japan; National Center for Global Health and Medicine, Department of Pediatrics, 1-21-1 Toyama, Shinjuku-ku, Tokyo 162-8655 Japan

**Keywords:** Childhood cancer, Survivor, Health-related quality of life

## Abstract

**Background:**

There are very few reliable and valid measures in Japan assessing health-related quality of life (HRQOL) in children with cancer. The present study aimed to develop a Japanese version of the Minneapolis-Manchester Quality of Life Survey of Health Adolescent Form (MMQL-AF), which is a measure for assessing the HRQOL of childhood cancer survivors, and investigate its reliability and validity.

**Methods:**

Participants were 141 children with cancer who had been off therapy for more than one year and 183 healthy controls. The reliability and internal consistency of the measure were assessed through test-retest methods using Cronbach’s coefficient alpha and intra-class correlation coefficients (ICCs). For validation of the measure, factorial validity, concurrent validity using the Japanese version of PedsQL 4.0 Generic Core Scales (PedsQL-J), and discriminant validity using comparisons between children with cancer and healthy controls were investigated.

**Results:**

Of the 46 items in the original version, 44 items were determined to comprise the Japanese version of the MMQL-AF. Cronbach’s coefficient alphas for each subscale were high ranging from 0.83 to 0.89. Test-retest reliability ranged between ICC 0.79 to 0.96. Investigation of concurrent validity using the PedsQL-J demonstrated strong correlations in physical functions and moderate correlations for other factors. A significant difference was observed between children with cancer and healthy controls.

**Conclusions:**

Thus, the Japanese version of the MMQL-AF served as a self-evaluation questionnaire that allowed for practical, comprehensive, and multidimensional measurement of HRQOL specific to childhood cancer survivors.

## Background

The treatment of childhood cancer has made remarkable progress with multidisciplinary treatment. A cure can be expected in more than 70–80% of children who have been diagnosed with childhood cancer [1-3]. However, many children are facing various late effects associated with the treatment directly and/or indirectly through long-term treatment, including chemotherapy, radiotherapy, and surgical therapy, in the course of overcoming childhood cancer, in addition to the disorders caused by the disease itself [4-6]. Late effects include impaired growth and development, disorders of the endocrine system such as gonadal dysfunction and hypothalamo-pituitary disorders accompanying impaired growth and development, cardiac/renal dysfunction, secondary cancer associated with treatment, intellectual impairment, and psychological disorders. The types and symptoms of late effects differ depending on the disease, therapy, the age the patient receiving the therapy, and sex; thus, any late effects can be a factor deteriorating the quality of life (QOL) of the children [7,8]. Even in children who recovered without physical late effects, many difficulties with the aspect of psychological or social QOL have been reported. For example, when entering school, employment, and into a marriage, they tend to suffer a psychological burden and social disadvantage, thus the decrease in QOL in these patients is a concern [9-11].

Developmental psychologists have noted that self-esteem has been a significant factor influencing identity formation during adolescence [12]. Furthermore, developmental psychologists have also conducted studies examining the association between QOL and self-esteem in child cancer survivors during adolescence [13-16].

With these points of view, it is essential to construct a follow-up support system focusing on maintaining or improving long-term QOL, including the process of growth and development after a cure, as well as during treatment, for children with cancer [17-19]. Thus, a study on health-related quality of life (HRQOL) in childhood cancer survivors is a top priority in order to optimize the treatment of children with cancer.

While measures for the assessment of the HRQOL are being developed for adults, in recent years, there have been both generic [20-22] and cancer specific [23,24] measures being developed in Japan. Additionally, there are very few measures for the assessment of the HRQOL for childhood cancer patients and survivors for which reliability and validity have been fully demonstrated [25-27], and there are none for survivors of specific cancers.

The Minneapolis-Manchester Quality of Life Survey of Health (MMQL) was designed as a comprehensive, multidimensional self-report instrument for measuring HRQOL among survivors of childhood cancer [28,29], and is frequently utilized in other countries [30-35].

The present study aimed to develop a Japanese version of a globally comparable HRQOL measurement for childhood cancer survivors and investigate its reliability and validity. The development of the Japanese version of this measure for evaluation will serve to assist the construction of a long-term follow-up support system in Japan for childhood cancer survivors.

## Methods

### Scale development

We obtained permission from Dr. Smita Bhatia to translate the Minneapolis-Manchester Quality of Life Survey of Health Adolescent Form (MMQL-AF) into Japanese using a standardized validation procedure. Two translators, an American who had been living in Japan for many years and was fluent in Japanese and the other, a Japanese who was fluent in English independently translated the MMQL-AF into Japanese. The translators and the authors discussed the translation and whether it was conceptually equivalent to the original English version. Then, a professional bilingual translator (English and Japanese) back translated the Japanese version of the MMQL-AF into English. There were only a few minor changes in the back translated Japanese version, and we conducted a pilot test on six children aged 13–18 years (mean age, 15.5 ± 1.71; 3 males, 3 females, 3 with cancer off therapy and 3 healthy controls). We asked children whether they understood the questions and noted the amount of time they needed to complete the questionnaire. There were no significant problems; therefore, we completed the Japanese version of MMQL-AF.

### Study population

#### Children with cancer

We recruited children who met the following eligibility criteria (according to the original study of the MMQL) from eight hospitals in Japan.

Eligibility criteria were as follows: 1) Participants aged ≥ 13 years and < 19 years. The age category for the Minneapolis-Manchester Quality of Life Survey of Health Adolescent Form (MMQL-AF) is between 13 and 20 years of age, while the age category for the Japanese version of the Pediatric Quality of Life Inventory (PedsQL-J), which was used as a validation scale in the present study, is 13–18 years of age. Therefore, the upper age limit of the present study was determined to be < 19 years of age; 2) Diagnosed with childhood cancer and off therapy for more than one year after completion of the treatment (in cases of recurrence, after completion of its treatment); 3) Informed consent for participation in the study was obtained from the parents/guardian as well as the children.

Exclusionary criteria were as follows: 1) Children diagnosed with cancer and still undergoing therapy; 2) Children whose mental and/or physical status did not allow for participation in the survey; 3) Children with a serious cognitive disorder; 4) Children with a serious chronic physical disease other than cancer; or 5) Any individuals whom the attending physician determined unsuitable for this study.

#### Healthy controls

We recruited children aged 13–18 years of age (with no history of cancer or any other chronic illness) from junior high school and high school in Tokyo.

### Procedure and measurement

The children for whom informed consent for participation in this study had been obtained from the children themselves and the parents/guardians were asked to respond to the following questionnaires:

#### Japanese version of Minneapolis-Manchester quality of life survey of health adolescent form (MMQL-AF)

Minneapolis-Manchester Quality of Life Survey of Health (MMQL) is a self-report questionnaire recognized globally and allows for practical, comprehensive, and multidimensional measurement of HRQOL specific to childhood cancer survivors. Depending on the developmental stage of the children, two versions of the questionnaire, i.e., for children aged 8–12 years (MMQL-Youth Form) [29] and for those aged 13–20 years (MMQL-Adolescent Form) [28] have been developed at each language level. In the present study, the MMQL-AF was used. MMQL-AF consists of 46 items and is composed of the following seven subscales—physical functioning (9 items); cognitive functioning (9 items); psychological functioning (9 items); body image (6 items); social functioning (6 items); outlook on life (3 items); and human relationships (4 items).

#### Japanese version of the pediatric quality of life inventory (PedsQL-J)

The Japanese version of the HRQOL scale for children, which was developed by Kobayashi et al. [25], was used in the present study for children ages 13–18 years. It consists of 23 items and the following four subscales: physical functioning (8 items); emotional functioning (5 items); social functioning (5 items); and school functioning (5 items). Additionally, its reliability and validity have been previously demonstrated. This scale is used as a standard for the investigation of the validity of the Japanese version of MMQL-AF, with approval from Dr. James W. Varni, the author of the original version, MAPI Research TRUST, the copyright holding organization, and Dr. Kyoko Kobayashi and Dr. Kiyoko Kamibeppu, the authors of the Japanese version.

#### Scale for the consciousness of self-affirmation

The scale for the Consciousness of Self-affirmation is a scale developed by Hiraishi [36,37], which focuses on psychological health during adolescence. This scale is used to discuss the influence of self-esteem specific to adolescents using a developmental perspective in order to deepen the psychological understanding of childhood cancer survivors during adolescence. This scale consists of 41 items in a questionnaire format divided into two fields, i.e., an intrapersonal field and an interpersonal field. The lower-level components for each field are as follows: Lower-level components for the intrapersonal field include self-acceptance (4 items); attitude of self-actualization (7 items); and sense of fulfillment (8 items). The intrapersonal field is related to psychological health in adolescence, especially self-esteem/self-image. Lower-level components for the interpersonal field include the following scales: self-closure/misanthropy (8 items); self-expression/interpersonal positivity (7 items); and sense of being evaluated/interpersonal tension (7 items). The interpersonal field is related to social competence, such as interpersonal tension, interpersonal distrust, or positive interpersonal attitude. This scale is also used as a standard for the investigation of the validity of the Japanese version of the MMQL-AF.

For healthy controls, a question assessing whether they had any chronic illness or disease that required long-term treatment in the hospital was added.

For children with cancer, information on the following items was obtained from the physician of the cooperating institution for the study: 1) Child’s sex; 2) Child’s age; 3) Name of the disease; 4) Date of diagnosis or date of start of treatment; 5) Date of completion of treatment; 6) History of recurrence (if yes, provide the date of the completion of treatment after recurrence); 7) History of surgery (If yes, provide the site of surgery); 8) History of chemotherapy; 9) History of radiotherapy (If yes, provide the site of irradiation and dosage); 10) History of hematopoietic stem cell transplantation (If yes, provide (a) type, (b) source of stem cell, (c) number of times); and 11) Experience of late effects.

### Conducting the survey

At cooperating institutions for the study, approval by the Institutional Review Board was obtained after a review, and approval by the head of the institution was obtained.

#### Children with cancer

Physicians selected prospective subjects based on the eligibility criteria of this study, explained the purpose and contents to the parents/guardians both orally and in writing, and obtained their consent.

In the explanation document, the term “disease that requires long-term treatment in the hospital” was used instead of “childhood cancer” as there may be some cases where the “childhood cancer” disease was kept a secret from the child.

After obtaining informed consent for participation in this survey from the parents/guardians, the physician explained the purpose and the contents of this study to the children both orally and with written documents.

When children agreed to participate in this study, they completed the survey form, which was a self-report questionnaire, without providing their name, and mailed it in to the researcher (MK).

#### Healthy controls

Junior high school and high school children and their parents/guardians received an explanatory pamphlet and questionnaire package. When children and the parents/guardians agreed to participate in the survey, children completed the questionnaire, without providing their name, and mailed it in to the researcher (MK).

### Test-retest reliability

Test-retest reliability was assessed at the National Cancer Center Hospital. Thirty children and their parents/guardians agreed to take a retest after two weeks. The questionnaires were mailed to the families two weeks after the initial assessments and were all returned within two weeks.

### Statistical analysis

Statistical analyses of the study were conducted using SPSS 20.0 J for Windows (SPSS, Inc., Chicago, IL) and the significance level was set at *p* < 0.05.

Factor analysis was used to confirm the structure of the subscales of the Japanese version of the MMQL-AF. Reliability was determined by internal consistency using Cronbach’s alpha and test-retest reliability using Spearman’s intra-class correlation coefficient (ICC). Internal consistency was considered good when Cronbach’s alpha exceeded 0.70 [38].

Concurrent validity was assessed by Spearman’s correlation coefficient between the MMQL-AF and PedsQL-J. Discriminate validity was determined by comparing the participant groups (children with cancer and healthy controls).

Feasibility was determined by the percentage of missing values and by the amount of time required to complete the questionnaires.

### Ethical considerations

The present study was approved by the Institutional Review Board at each participating institution and Tokyo Medical Dental University.

## Results

### Subject characteristics

The present study included 141 children with cancer who completed treatment at least one year prior and 183 healthy controls with no previous history of cancer or other chronic diseases. Table 1 presents the characteristics of the study population. The mean time after completion of treatment was 6.23 years (ranging from 1.0 to 15.25 years) for the children with cancer group. There were no significant differences between the groups with regard to age (*t*(322) = 0.12, *p* = 0.91, ns) and sex (*χ*^*2*^(1) = 4.08, *p* = 0.13, ns). A total of 88 subjects (62.4%) in the children with cancer group had hematological diseases, while the remaining subjects had solid tumors.

**Table 1:** **Descriptive statistics of the study population**

	**Children with cancer off therapy (n = 141)**		**Healthy controls (n = 183)**	
	**n**	**%**	**n**	**%**
Age, years				
Mean (SD)	15.75 (1.79)		15.73 (2.03)	
Sex				
Male	81	57.4	86	47.0
Female	60	42.6	96	52.5
Unknown			1	.5
Diagnosis				
Acute lymphoblastic leukemia	46	32.6		-
Acute myeloid leukemia	11	7.8		-
Other leukemia	8	5.7		-
Non-Hodgkin lymphoma	23	16.3		-
Osteosarcoma	15	10.6		-
Neuroblastoma	6	4.3		-
Rhabdomyosarcoma	5	3.5		-
Brain tumor	5	3.5		-
Hepatoblastoma	3	2.1		-
Wilms tumor	1	.8		-
Other cancer	18	12.8		-

n: number of individuals, SD: standard deviation.

### Structure of the MMQL-AF

To confirm the scale structure of the Japanese version of the MMQL-AF, factor analysis (principal factor analysis) was conducted using the 46 items. In this analysis, a factor loading value ≥ 0.40 was considered sufficiently high. Based on changes in Eigenvalues and the interpretability of factors, it appeared that a 6-factor structure was appropriate rather than the 7-factor structure of the original version. After excluding two items with no sufficient factor loadings (“Feeling inferior to most people” and “Prefer to watch rather than take part in games and sports”), factor analysis was again conducted with the remaining 44 items on a hypothesized 6-factor structure. Table 2 presents the final factor pattern and inter-factor correlations after using a Promax rotation. The social functioning and intimate relations factors in the original version were combined into a single factor. It should be noted that the cumulative contribution ratio before rotation was 56.83%.

**Table 2:** **Factor analysis of the Japanese version of the MMQL-AF**

	**Factor 1**	**Factor 2**	**Factor 3**	**Factor 4**	**Factor 5**	**Factor 6**
**Factor 1: Social functioning & intimate relations**						
Have many close friends	**.89**	.00	.00	-.09	-.05	.03
Getting along well with people their age	**.85**	-.01	-.01	.04	-.09	-.04
Having a lot in common with their friends	**.75**	.04	-.12	-.09	-.09	.05
Being together with other people gives them a good feeling	**.75**	-.09	-.04	-.03	-.03	.11
Having similar hobbies and interests to people their age	**.73**	-.07	.00	-.07	.08	.02
Feel left out in groups of people their age	**-.63**	-.17	-.04	-.05	-.06	.06
Difficulty in making friends	**-.57**	-.07	-.08	-.16	-.11	.13
Find it easy to have an intimate relationship	**.53**	.04	.05	.04	.20	-.03
Believing that people like to be with them	**.49**	-.08	.15	.06	-.11	.11
Feel confident when they are with people of opposite sex	**.48**	-.05	.00	.07	.10	-.03
**Factor 2: Psychological functioning**						
Feeling sad	-.01	**.88**	.03	-.03	-.08	.01
Feeling nervous or anxious	.01	**.88**	.05	-.07	.02	-.07
Feeling frightened	-.07	**.87**	.09	-.04	-.01	-.01
Feeling lonely	.13	**.80**	.05	-.10	.01	-.12
Feeling angry	.05	**.73**	.01	.02	-.11	.04
Worried about things in general	.00	**.60**	-.15	-.02	.11	.09
Worried about dying	-.08	**.50**	.02	-.13	.06	.06
Feeling tired during the day	.03	**.49**	-.04	.34	.02	.03
Worried about their health	-.18	**.41**	-.12	.15	.18	.14
**Factor 3: Cognitive functioning**						
Difficulty with school work compared with others in class	-.03	.05	**.80**	-.10	.01	-.03
Homework or study is hard for them	.06	.03	**-.77**	-.11	.06	-.06
Difficulty in concentrating at school	.07	.01	**-.70**	-.12	.06	-.08
Difficulty in concentrating at work or school	-.01	-.06	**.69**	-.03	.20	.00
Needing more help with school work than others in class	.00	.06	**-.66**	-.07	.06	-.14
Difficulty with math or calculations	.11	-.08	**.64**	-.09	.16	-.09
Difficulty with reading or writing	-.03	.12	**.62**	-.04	-.03	-.13
Difficulty with remembering things at school/college	.05	.07	**.61**	.02	-.03	.05
Difficulty concentrating at other times (computer/games/playing cards/reading)	-.05	-.03	**-.43**	.03	.08	-.14
**Factor 4: Physical functioning**						
Unable to keep up with others of their age when taking part in sports	-.05	-.14	.03	**.80**	-.05	-.04
Unable to do many activities because of health	.13	.02	.01	**-.80**	-.06	.10
Unable to do many activities because of arms or legs	-.04	.06	.01	**-.72**	.05	.14
Have a lot of energy for running or sports	.18	-.06	-.03	**.66**	-.04	.06
Feel strong and healthy	.04	-.01	-.01	**-.59**	-.14	-.13
Need time during day to rest	.00	-.11	-.24	**-.50**	.11	-.01
Have a lot of energy	.15	.16	-.16	**.49**	-.02	.24
**Factor 5: Body image**						
Liking their body the way it is	-.08	-.02	-.02	.02	**.74**	.15
Feelings about their body development	.01	.06	-.06	.01	**.72**	.03
Being happy about the way they look	.11	-.02	.04	-.24	**.68**	.11
Feeling uncomfortable about the way their body is developing	.01	.05	.01	-.26	**-.68**	.07
Being satisfied about their weight	-.04	.05	.00	-.19	**.66**	.00
Feeling that others think that their body is poorly developed	-.03	-.04	-.09	-.16	**-.59**	.18
**Factor 6: Outlook on life**						
Satisfied with the current life situation	.02	.01	.04	-.02	-.04	**.83**
Happy with life in general	-.03	.06	.06	.00	.08	**.77**
Happy with the way things are	.10	.01	.03	-.12	.07	**.65**
Factor correlation	Factor 1	Factor 2	Factor 3	Factor 4	Factor 5	Factor 6
Factor 1	-	.56	.47	.40	.43	.42
Factor 2		-	.44	.37	.47	.50
Factor 3			-	.36	.36	.37
Factor 4				-	.24	.34
Factor 5					-	.43
Factor 6						-
α	.89	.89	.88	.83	.83	.84

Extraction method is principle factor analysis using Promax rotation with Kaiser normalization.

Factor loadings greater than 0.40 are shown in boldface.

MMQL-AF: Minneapolis-Manchester Quality of Life Survey of Health - Adolescent Form., α: Cronbach’s coefficient.

### Reliability

To assess the internal consistency of the Japanese version of the MMQL-AF, Cronbach’s alpha coefficient was calculated. Results showed sufficient Cronbach’s alpha coefficients for factors one to six (0.83 to 0.89, respectively) (see Table 2).

Table 3 presents the test-retest reliability analysis of the Japanese version of the MMQL-AF. Excellent ICC values > 0.90 were obtained with the exception of psychological functioning, for which the ICC value was good to fair at 0.79.

**Table 3:** **Test-retest reliability of the Japanese version of the MMQL-AF (n = 30)**

	**First**		**Second**		**ICC**
**MMQL-AF questionnaire**	**Mean**	**SD**	**Mean**	**SD**	
Physical functioning	15.41	6.01	14.90	6.07	.96^**^
Psychological functioning	19.17	6.83	17.97	5.70	.79^**^
Body image	15.33	4.39	15.20	4.45	.90^**^
Social functioning & intimate relations	19.86	6.39	20.28	7.33	.90^**^
Cognitive functioning	15.45	5.96	15.21	6.22	.92^**^
Outlook on life	6.30	2.98	5.97	3.07	.90^**^

MMQL-AF: Minneapolis-Manchester Quality of Life Survey of Health-Adolescent Form, n: number of individuals, SD: standard deviation, ICC: intraclass correlation coefficient. **p < 0.01.

### Validity

To assess the concurrent validity of the Japanese version of the MMQL-AF, Spearman’s correlation coefficients were calculated between subscale scores for the Japanese version of the MMQL-AF and the PedsQL-J (Table 4). The physical functioning scale on the MMQL-AF was strongly correlated with the physical functioning scale and weakly correlated with each of the other scales on the PedsQL-J. Each of the psychological, social functioning and intimate relations, and outlook on life scales of the MMQL-AF was moderately correlated with each of the physical, emotional, and social functioning scales and weakly correlated with the school functioning scale of the PedsQL-J. The body image scale on the MMQL-AF was weakly correlated with all scales on the PedsQL-J. The cognitive functioning scale of the MMQL-AF was moderately correlated with all scales on the PedsQL-J.

**Table 4:** **Spearman’s correlation coefficients between the Japanese version of the MMQL-AF and the PedsQL-J (n = 324)**

	**PedsQL-J**			
**MMQL-AF questionnaire**	**Physical functioning**	**Emotional functioning**	**Social functioning**	**School functioning**
Physical functioning	.720^**^	.345^**^	.361^**^	.353^**^
Psychological functioning	.454^**^	.593^**^	.527^**^	.359^**^
Body image	.381^**^	.390^**^	.385^**^	.307^**^
Social functioning & intimate relations	.437^**^	.460^**^	.649^**^	.286^**^
Cognitive functioning	.516^**^	.413^**^	.462^**^	.460^**^
Outlook on life	.424^**^	.472^**^	.424^**^	.366^**^

MMQL-AF: Minneapolis-Manchester Quality of Life Survey of Health-Adolescent Form, PedsQL-J: the Japanese version of the Pediatric Quality of Life Inventory, n: number of individuals. ^**^ *p* < 0.01.

To assess the discriminant validity of the Japanese version of the MMQL-AF, scores for each subscale on the MMQL-AF were compared between children with cancer and healthy controls (Table 5). Results indicated that the children with cancer had significantly (<0.01) higher scores for the physical functioning scale compared to the healthy controls. Compared with the healthy controls, the physical functioning scale on the PedsQL-J, which was strongly correlated with the physical functioning scale on the MMQL-AF (Table 4), also showed significantly (<0.01) higher scores among children with cancer (Table 6). Comparisons of the scores for each subscale on the MMQL-AF between the children with cancer and the healthy controls demonstrated that children with cancer showed significantly (<0.05) higher scores on the social functioning and intimate relations and cognitive functioning scales compared to the healthy controls (Table 5). For the consciousness of self-affirmation scale, significant differences in the scores were found between children with cancer and the healthy controls on both the interpersonal and intrapersonal fields (<0.05, Table 7).

**Table 5:** **Discriminant validity involving known group analysis for the Japanese version of the MMQL-AF**

	**Children with cancer off therapy (n = 141)**		**Healthy controls (n = 183)**		***t***	***p*** **value**
**MMQL-AF questionnaire**	**Mean**	**SD**	**Mean**	**SD**		
Physical functioning	13.62	5.36	8.92	2.47	9.46	< 0.01
Psychological functioning	20.32	7.87	19.59	7.48	.84	ns
Body image	15.30	4.56	14.98	3.98	.67	ns
Social functioning & intimate relations	20.37	7.76	18.67	5.96	2.13	< 0.05
Cognitive functioning	15.47	5.73	13.95	5.16	2.48	< 0.05
Outlook on life	6.15	2.81	5.93	2.48	.74	ns

MMQL-AF: Minneapolis-Manchester Quality of Life Survey of Health-Adolescent Form, n: number of individuals, SD: standard deviation, *t*: *t* value.

**Table 6:** **Discriminant validity involving known group analysis for the PedsQL-J**

	**Children with cancer off therapy (n = 141)**		**Healthy controls (n = 181)**		***t***	***p*** **value**
**PedsQL-J**	**Mean**	**SD**	**Mean**	**SD**		
Physical functioning	5.15	5.17	2.22	3.76	5.66	< 0.01
Emotional functioning	5.35	4.06	5.27	3.61	.18	ns
Social functioning	2.85	3.50	2.52	2.80	.92	ns
School functioning	4.45	3.10	3.89	2.71	1.69	ns

PedsQL-J: the Japanese version of the Pediatric Quality of Life Inventory, n: number of individuals, SD: standard deviation, *t*: *t* value.

**Table 7:** **Discriminant validity involving known group analysis of the scale for the consciousness of self-affirmation**

	**Children with cancer off therapy (n = 140)**		**Healthy controls (n = 181)**		***t***	***p*** **value**
**Scale for the consciousness of self-affirmation**	**Mean**	**SD**	**Mean**	**SD**		
Intrapersonal field^a^	46.85	7.32	44.87	9.13	2.15	< 0.05
Interpersonal field^b^	72.05	10.59	74.35	9.83	1.98	< 0.05

n: number of individuals, SD: standard deviation, *t*: *t* value.

^a^self-acceptance, attitude of self-actualization, sense of fulfillment.

^b^self-closure/misanthropy, self-expression/interpersonal positivity, sense of being evaluated/interpersonal tension.

### Feasibility

The percentage of missing values in the present study was 0.40%. During pilot testing, all the items on the MMQL-AF were completed in 6–12 minutes.

## Discussion

In Japan, there are estimated to be over several million childhood cancer survivors, and the number of childhood cancer survivors is reported to be increasing at a rate of ≥ 1400 individuals per year [39]. In addition to complications with intensive treatment, childhood cancer is associated with problems following treatment during the growth and development period, such as failure to thrive/developmental disorders, endocrine disorders, organ damage, gonadal disturbances, high brain dysfunction, and second primary cancers, even after a cure is achieved. To provide long-term support for childhood cancer survivors in their daily lives and school attendance/employment after completion of treatment, long-term follow-up should involve HRQOL assessments and appropriate measures from a multidimensional perspective. These should include not only the medical-physical aspects, but also the psychosocial aspects according to the developmental process. Although both generic and disease-specific multidimensional measures for children and adolescents have been developed in Western countries, only limited HRQOL measures for children and adolescents with established reliability and validity are available in Japan [25-27].

The Pediatric Quality of Life Inventory (PedsQL) is a modular instrument designed to measure HRQOL in children and adolescents aged 2–18 years. The Japanese version of the PedsQL 4.0 Generic Core Scales was standardized by Kobayashi et al. [25] and was used to assess the concurrent validity in the present study. In 2011, the Japanese version of the PedsQL 3.0 Cancer Modular was standardized by Tsuji et al. [27]. The PedsQL 3.0 Cancer Modular is designed to assess a wide age range of 2–18 years with proxy assessments by guardians used for younger children. It is a measure designed for children with cancer, although the standardization of the Japanese version included individuals who had completed treatment at least a year prior.

The Japanese version of the MMQL-AF is a self-administered HRQOL instrument specific to childhood cancer survivors aged 13–18 years (adolescents falling under the categories of junior high and high school students in Japan) who have completed treatment.

Factor analysis of the Japanese version of the MMQL-AF showed different results from those obtained for the original version of the MMQL-AF [28]. First, two items with non-sufficient factor loadings were excluded. Second, the social functioning and intimate relations scales in the original version were combined into a single factor. Although the intimate relations scale was originally composed of four items (“Difficulty in making friends,” “Feel left out in groups of people their age,” “Feel confident when they are with people of the opposite sex,” and “Find it easy to have an intimate relationship”) [28], the subsequent final version of the MMQL-AF has “Difficulty in making friends” and “Feel left out in groups of people their age” included in the social functioning subscale and “Feel confident when they are with people of the opposite sex” and “Find it easy to have an intimate relationship” included in the intimate relations subscale. Considering that the instructions appearing on the intimate relations subscale on the questionnaire state “about close relationships with other people,” combining this subscale with the social functioning factor, which asks about “the relationship people have with their family,” is acceptable and rather natural for responders.

Assessment of the reliability of the Japanese version of the MMQL-AF found that all factors had Cronbach’s coefficient alpha values > 0.80, indicating evidence of high internal consistency.

For test-retest reliability, excellent ICC values > 0.90 were obtained with the exception of psychological functioning, for which the ICC value was good to fair at 0.79. Based on these results, it can be considered that the Japanese version of the MMQL-AF is a highly reliable measure.

For validation of the Japanese version of the MMQL-AF, Spearman’s correlation coefficients were calculated between subscale scores for the Japanese version of the MMQL-AF and the PedsQL-J to assess the concurrent validity of the Japanese version of the MMQL-AF. Scores for all subscales in the MMQL-AF were significantly correlated with the scores for all subscales on the PedsQL-J. In particular, the physical functioning subscale on the Japanese version of the MMQL-AF was very strongly correlated with the physical functioning subscale on the PedsQL-J, and each of the psychological functioning, social functioning and intimate relations, and outlook on life scales on the MMQL-AF was moderately correlated with each of the physical, emotional, and social functioning scales of the PedsQL-J. The body image scale of the MMQL-AF was only weakly correlated with any scale of the PedsQL-J. Based on these results, the Japanese version of the MMQL-AF can be considered a structurally valid HRQOL instrument. The body image subscale can be considered a subscale characteristic of the MMQL-AF.

To assess the discriminant validity of the Japanese version of the MMQL-AF, scores for each subscale on the MMQL-AF were compared between children with cancer and healthy controls. Results indicated that children with cancer had significantly higher scores for the physical functioning subscale compared to the healthy controls, indicating that children with cancer tended to feel limitations during daytime activities. Compared to healthy controls, the physical functioning subscale on the PedsQL-J, which was strongly correlated with the physical functioning subscale on the MMQL-AF, also showed significantly (<0.01) higher scores among the children with cancer, indicating that the physical functioning subscale on the MMQL-AF represented a measure of physical function in children with cancer.

Comparisons of the scores for each subscale on the MMQL-AF between the children with cancer and the healthy controls demonstrated that children with cancer showed significantly higher scores for the social functioning and intimate relations subscales compared to healthy controls, indicating that children with cancer tended to have greater anxiety about relations with others. The children with cancer also showed significantly higher scores for the cognitive functioning subscale compared with the healthy controls, indicating that children with cancer tended to have a greater feeling of lack of fulfillment in school or work. The scale for the consciousness of self-affirmation is a measure related to psychological health during adolescence, which consists of the intrapersonal field containing the three components of self-acceptance, attitude of self-actualization, and sense of fulfillment and the interpersonal field containing the three components of self-closure/misanthropy, self-expression/positive interpersonal attitude, and sense of being evaluated/interpersonal tension.

No differences in any component were found between children with cancer and healthy controls, although there were significant differences between children with cancer and the healthy controls with regard to scores for both the intrapersonal field and the interpersonal field. The intrapersonal field was related to psychological health during adolescence, especially self-esteem/self-image, and higher scores for this field indicate lower self-esteem/self-image. In contrast, the interpersonal field was related to social competence, such as interpersonal tension, interpersonal distrust, or positive interpersonal attitude and lower scores for this field indicate lower social competence. Comparisons of scores for each subscale on the MMQL-AF between the children with cancer and the healthy controls showed a significantly higher score for the social functioning and intimate relations subscales among children with cancer compared to the healthy controls. Additionally, the observed tendency for children with cancer to have greater anxiety about relations with others may be explained by lower social competence among them compared to healthy controls as evidenced by the interpersonal field of the scale for the consciousness of self-affirmation. In addition, a significantly higher score for the cognitive functioning subscale on the MMQL-AF was obtained among the children with cancer compared with the healthy controls, and the observed tendency for children with cancer to have a greater feeling of lack of fulfillment in school or work may be explained by a tendency towards lower self-esteem/self-image among children with cancer compared to the healthy controls as evidenced in the intrapersonal field of the scale for the consciousness of self-affirmation. There were no significant differences between children with cancer and healthy controls with regard to the scores for any of the psychological functioning, body image, and outlook on life subscales.

The percentage of missing values in the present study was 0.40%. During pilot testing, all the items on the MMQL-AF were completed in 6–12 minutes, indicating acceptable feasibility of the Japanese version of the MMQL-AF.

### Limitations and future direction

The main purpose of the present study was to examine the validity and reliability of the Japanese version of the MMQL-AF; however, the present study did not examine the scale’s responsiveness to change. Responsiveness to change is an important factor for predicting how and why children with cancer will change over time. Thus, further examination of the measure and its responsiveness to change is warranted in future studies.

As the number of participants with each cancer type was limited in this study, future researchers may want to conduct more elaborate procedures in order to determine the effects and impact of each cancer type within the recovery process. The present study did find significant differences between the hematological disease group and the solid tumor group, as well as between the male and female groups. Children in the solid tumor group tended to have restricted mobility in physical functioning (t(140) = −3.06, *p* < 0.01), and males tended to display negative emotions in psychological functioning (t(140) = −2.80, *p* < 0.01).

## Conclusion

The results of the present study showed evidence for the reliability and validity of the MMQL-AF as a comprehensive, multidimensional self-report instrument for measuring HQOL among adolescent survivors of childhood cancer. As Dr. Smita Bhatia mentioned in the original study, the instrument has been tested in a random sample of healthy children who had no chronic illness, and thus provides normative data that can be used in future studies for comparing HRQOL in various populations.

## Abbreviations

HRQOL: Health-related quality of life
ICC: Intra-class correlation coefficient
MMQL: Minneapolis-Manchester quality of life survey of health
MMQL-AF: Minneapolis-Manchester quality of life survey of health adolescent form
PedsQL: Pediatric quality of life inventory
PedsQL-J: Japanese version of PedsQL
QOL: Quality of life
SD: Standard deviation
SPSS: Statistical package for social science
